# Evaluation of the 2007 WHO Guideline to Improve the Diagnosis of Tuberculosis in Ambulatory HIV-Positive Adults

**DOI:** 10.1371/journal.pone.0018502

**Published:** 2011-04-06

**Authors:** Olivier Koole, Sopheak Thai, Kim Eam Khun, Reaksmey Pe, Johan van Griensven, Ludwig Apers, Jef Van den Ende, Tan Eang Mao, Lutgarde Lynen

**Affiliations:** 1 Institute of Tropical Medicine, Antwerp, Belgium; 2 Sihanouk Hospital Center of HOPE (SHCH), Phnom Penh, Cambodia; 3 National Center for TB and Leprosy Control (CENAT), Phnom Penh, Cambodia; McGill University, Canada

## Abstract

**Background:**

In 2007 WHO issued a guideline to improve the diagnosis of smear-negative and extrapulmonary tuberculosis (EPTB) in HIV-positive patients. This guideline relies heavily on the acceptance of HIV-testing and availability of chest X-rays.

**Methods and Findings:**

Cohort study of TB suspects in four tuberculosis (TB) clinics in Phnom Penh, Cambodia. We assessed the operational performance of the guideline, the incremental yield of investigations, and the diagnostic accuracy for smear-negative tuberculosis in HIV-positive patients using culture positivity as reference standard. 1,147 (68.9%) of 1,665 TB suspects presented with unknown HIV status, 1,124 (98.0%) agreed to be tested, 79 (7.0%) were HIV-positive. Compliance with the guideline for chest X-rays and sputum culture requests was 97.1% and 98.3% respectively. Only 35 of 79 HIV-positive patients (44.3%) with a chest X-ray suggestive of TB started TB treatment within 10 days. 105 of 442 HIV-positive TB suspects started TB treatment (56.2% smear-negative pulmonary TB (PTB), 28.6% smear-positive PTB, 15.2% EPTB). The median time to TB treatment initiation was 5 days (IQR: 2–13 days), ranging from 2 days (IQR: 1–11.5 days) for EPTB, over 2.5 days (IQR: 1–4 days) for smear-positive PTB to 9 days (IQR: 3–17 days) for smear-negative PTB. Among the 34 smear-negative TB patients with a confirmed diagnosis, the incremental yield of chest X-ray, clinical suspicion or abdominal ultrasound, and culture was 41.2%, 17.6% and 41.2% respectively. The sensitivity and specificity of the algorithm to diagnose smear-negative TB in HIV-positive TB suspects was 58.8% (95%CI: 42.2%–73.6%) and 79.4% (95%CI: 74.8%–82.4%) respectively.

**Conclusions:**

Pending point-of-care rapid diagnostic tests for TB disease, diagnostic algorithms are needed. The diagnostic accuracy of the 2007 WHO guideline to diagnose smear-negative TB is acceptable. There is, however, reluctance to comply with the guideline in terms of immediate treatment initiation.

## Introduction

The World Health Organization (WHO) has classified Cambodia as one of the 22 high burden countries with tuberculosis (TB) in the world. [1] In 2007 the TB incidence rate of all forms was estimated at 495/100,000 inhabitants per year, with estimated death rates of 77/100,000 and 13/100,000 per year among HIV-positive and HIV-negative populations respectively. [2] In 2006 the reported HIV prevalence fell to an estimated 0.9% among the adult population (15–49 years) from 1.9% in 2003. [3] In 2007, the HIV-prevalence among TB patients was estimated to be 7.8%. [4]

Rates of smear-negative pulmonary and extrapulmonary TB have been rising in countries with HIV epidemics. [5], [6] HIV-positive TB patients experience higher mortality rates than HIV-negative TB patients, especially those with smear-negative pulmonary or extrapulmonary tuberculosis. [7]–[10] Delayed TB diagnosis may be an important contributor to this excess mortality in HIV-infected persons. [7], [11]–[13]

Diagnosis of smear-negative and extrapulmonary tuberculosis is challenging and hampered by the lack of simple, rapid and accurate diagnostic tests. [14] Autopsy studies have found disseminated TB in 40–54% of HIV-infected people in HIV prevalent countries, many of whom were undiagnosed prior to death. [15]–[18]

In the absence of a rapid diagnostic test several diagnostic algorithms have been recommended. [19] In 2007, a WHO international expert committee issued a new guideline to improve the diagnosis of TB in HIV-positive patients in HIV prevalent settings. [20] The new guideline was in the first place designed to reduce the delay in diagnosing TB in HIV-positive patients and in the second place to include a more sensitive tool than direct microscopy. For that reason new investigation means were added (culture), and existing tools were allowed to be carried out earlier in the diagnostic pathway (X-ray), and this after all suspects were categorized into two groups: HIV-positive and HIV-negative patients. It was recognized by the expert team that this strategy would increase the sensitivity and compromise the specificity, but this was considered the necessary price to reduce mortality in the HIV-positive TB patients. This new guideline is based on expert opinion and relies heavily on the acceptance of an HIV-test and the availability of chest X-rays. In this cohort study we assessed the operational performance (acceptability, feasibility and compliance) of the WHO 2007 guideline in ambulatory TB suspects in four Cambodian TB clinics with a variable proportion of HIV-positive patients and we evaluated its performance to diagnose smear-negative TB in HIV-positive patients.

## Methods

### Settings

The study was conducted in four sites in Phnom Penh, Cambodia: the TB clinic of the National Center for Tuberculosis and Leprosy Control (CENAT) and the Pochentong District Hospital; the TB clinic of the Sihanouk Hospital Centre of Hope (SHCH), a NGO referral hospital, and the TB clinic of Chey Chumneas (CCN) Provincial Hospital. The latter two health structures have a higher proportion of HIV-positive patients as they are known as HIV treatment centers providing antiretroviral treatment.

### Inclusion and exclusion criteria

**Figure 1:**
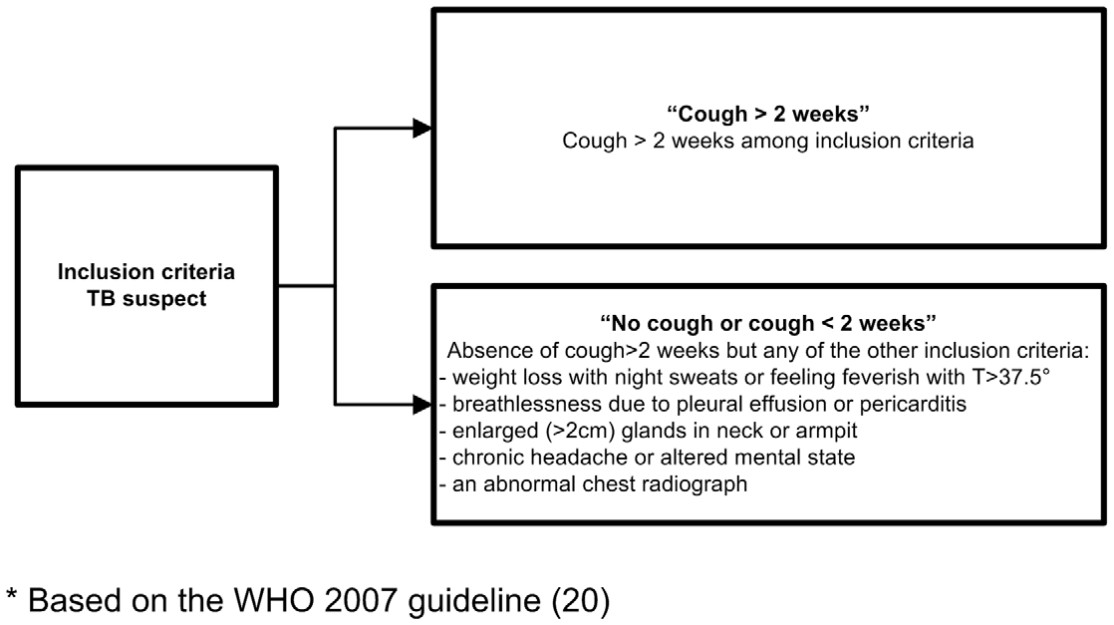
Inclusion criteria and screening questions for TB suspects*.

Adult patients (≥18 years old) were enrolled between July 2008 and July 2009. Based on the clinical characteristics suggestive of TB as described in the WHO diagnostic algorithm [20], TB suspects were classified in two groups: 1) cough for more than 2 weeks referred to as the “cough>2 weeks” group, or 2) absence of cough more than 2 weeks but any of the following symptoms: weight loss with night sweats or feeling feverish with T>37.5°, breathlessness due to pleural effusion or pericarditis, enlarged (>2 cm) glands in neck or armpit, chronic headache or altered mental state, or an abnormal chest radiograph, hereafter referred to as “no cough or cough <2 weeks” (**Figure 1**).

Patients with danger signs (defined by the WHO guideline as respiratory rate >30/minute, fever >39°C, pulse rate >120/minute and unable to walk unaided) should follow a different algorithm and were not the scope of this study, and thus excluded.

### Flow of patients

**Figure 2:**
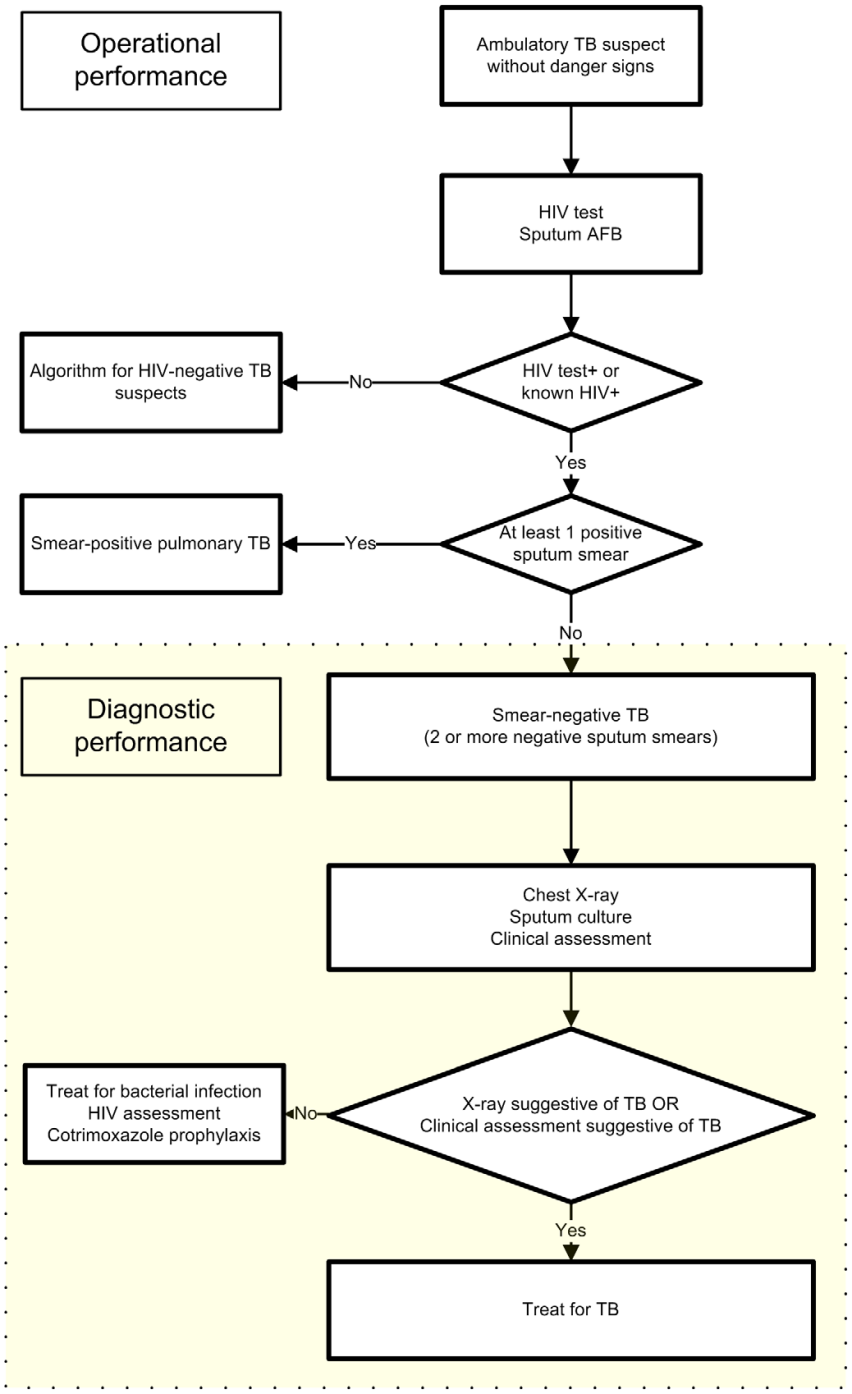
WHO algorithm for the diagnosis of tuberculosis in ambulatory HIV-positive patients.

All TB suspects with unknown HIV status were counselled and tested for HIV on the first day together with collection of a sputum sample for direct microscopic examination. Because of the low HIV prevalence in Cambodia, patients with a documented negative HIV test result less than a year old were considered HIV-negative (to avoid repeated HIV testing). For HIV-negative patients the national TB guideline based on the WHO 2003 guideline [21] was followed, for HIV-positive patients or patients with strong clinical suspicion of HIV the WHO 2007 guideline (**Figure 2**). [20] In the latter group a patient with one smear examination positive for acid-fast bacilli (AFB) was defined as smear-positive pulmonary tuberculosis. If the sputum was found to be smear-negative (at least 2 smears negative), a chest radiograph was requested. When it was suggestive of tuberculosis or when the clinical assessment was suggestive of TB (lymphadenitis or abnormal abdominal ultrasound) the patient had to be treated as smear-negative pulmonary or extrapulmonary tuberculosis without an antibiotic trial.

Abdominal ultrasound was performed in one setting (SHCH), though not systematically.

All TB patients were followed up until they finished TB treatment, unless they were transferred out, lost to follow-up (LTFU) or died.

### Laboratory methods

HIV testing was done on the day of presentation following the national HIV testing protocol. Sputum examinations were done on the day of presentation (on the spot) and on the next morning sample (morning and on the spot). SHCH and CENAT used auramine stain (positive smears confirmed by Ziehl-Neelsen), while the other centres used Ziehl-Neelsen (ZN). Mycobacterial culture of sputum was done for all HIV-positive TB suspects. The second sputum (morning) was stained with ZN at the site and subsequently sent for TB culture at CENAT. Solid media cultures (Ogawa) were used. In SHCH the third on spot specimen of HIV-positive TB suspects was also cultured using Löwenstein-Jensen medium.

### Imaging studies

For each chest X-ray a standardized form was filled out by the treating physician. The findings were summarized as normal, abnormal suggestive of TB, abnormal not suggestive of TB, and unknown or not available. All chest X-rays were later reviewed by 2 expert radiologists who were blinded to the patient' clinical condition. The inter-observer agreement was assessed by estimating pairwise kappa statistics. The values of kappa were classified according to the method described by Landis [22] for the distinction between normal and abnormal in all X-rays, and the distinction between TB and non-TB in X-rays defined as abnormal by both readers.

### Data management and analysis

The doctors and nurses of the study sites were trained in the use of the guideline and specific data-collection tools. Data were entered centrally in a research database (Microsoft Access 2003).

Compliance with the diagnostic algorithm and with the treatment recommendation was used to assess the operational performance of the guideline. Compliance with the treatment part of the WHO guideline was pre-specified in the protocol as “having started TB treatment within 10 days of presentation” (taking into account delays in results and appointments) in patients with two negative sputum smears and a chest radiograph suggestive of TB or clinical signs suggestive of TB.

The incremental yield of the different investigations was defined as the proportion of patients additionally diagnosed by X-ray if smear-negative, by clinical suspicion or abdominal ultrasound in smear-negatives without an X-ray suggestive of TB, and ultimately by culture if all of the above were negative. We assessed the incremental yield among HIV-positive patients treated for TB (decision by a clinician to treat with a full course of anti-TB chemotherapy with or without confirmation), and among confirmed smear-negative TB patients (using culture positivity at CENAT or SHCH for *Mycobacterium tuberculosis* as reference standard).

Time to TB treatment initiation was calculated for all patients treated for TB as the time between first presentation and start of TB treatment at the clinic. The outcomes of all TB patients were classified following standard WHO definitions [23] and retrieved using the national TB registers at CENAT.

The diagnostic performance of the WHO 2007 guideline to diagnose smear-negative TB was assessed by excluding smear positive PTB cases from the analysis. We calculated the sensitivity and specificity, and positive and negative predictive value of the WHO 2007 diagnostic algorithm using culture positivity at CENAT or SHCH for *Mycobacterium tuberculosis* as reference standard. Patients without X-ray results or culture results were excluded from this analysis. Confidence intervals around sensitivity and specificity were calculated using the Wilson's method. Appropriateness of pooling the observed sensitivities and specificities over different sites was tested using logistic regression models.

All statistical analyses were performed using Stata software, version 11.1. (Stata Corporation, College Station, TX, USA).

### Ethics statement

The study protocol, data collection forms and informed consent procedure were approved by the Research Ethics Review Committee of WHO, by the Institutional Review Board of the Institute of Tropical Medicine in Antwerp, by the Ethics Committee University Hospital Antwerp, and by the National Ethics Committee in Cambodia. Only patients who gave written informed consent were enrolled in the study.

### Role of the funding source

The study was funded by WHO (WHO registry file number T9-181-298), with additional funding from the Belgian Development cooperation (DGDC) through a framework agreement with the Institute of Tropical Medicine in Antwerp (institutional strengthening project 920800) and the Belgian National Association against Tuberculosis (BNBTTB).

The funders had no role in study design, data collection and analysis, decision to publish, or preparation of the manuscript.

## Results

A total of 1,677 TB suspects were enrolled. Of these patients, 12 were excluded from further analysis: 10 patients were below 18 years at presentation, and 2 patients had already started TB treatment at presentation.

### Patient characteristics

1,665 patients were included at the four sites (**Table 1**). The median age of patients enrolled was 37 years (range, 18–86), and 844 (50.7%) were male. The majority of TB suspects presented with “cough more than two weeks” (86.5%), ranging from 72.4% at Chey Chumneas (CCN) Provincial Hospital to 99.6% at Pochentong District Hospital. TB suspects with “no cough or cough <2 weeks” mainly presented at SHCH and CCN Provincial Hospital. The main entry points for TB suspects with “no cough or cough <2 weeks” were weight loss (80.9%) and/or chronic headache/altered mental state (48.4%). 442 (26.5%) TB suspects were HIV positive. The HIV prevalence varied greatly between the sites ranging from 2.1% at CENAT to 46.9% at CCN and 55.4% at SHCH.

Among the 442 HIV-positive TB suspects 312 (70.6%) presented as suspects with “cough > 2 weeks” and 130 (29.4%) with “no cough or cough < 2 weeks”.

**Table 1:** Characteristics and entry points of analyzed TB suspects.

	TB suspects	Entry-point		HIV+ TB suspects
		Cough > 2 weeks	No cough or cough <2 weeks*	
**Total**: n (%)	1665 (100.0)	1440 (86.5)	225 (13.5)	442 (26.5)
Age: years, median (range)	37 (18–86)	38 (18–86)	36 (18–73)	35 (18–69)
Male sex: n (%)	844 (50.7)	760 (52.8)	84 (37.3)	190 (43.0)
Location: n (%)				
Sihanouk Hospital Centre of Hope	478 (28.7)	361 (75.5)	117 (24.5)	265 (55.4)
National Center for Tuberculosis and Leprosy Control	615 (36.9)	598 (97.2)	17 (2.8)	13 (2.1)
Chey Chumneas Provincial Hospital	326 (19.6)	236 (72.4)	90 (27.6)	153 (46.9)
Pochentong District Hospital	246 (14.8)	245 (99.6)	1 (0.4)	11 (4.5)

*Patients with no cough or cough <2 weeks had other inclusion criteria.

### Operational performance of the WHO algorithm

#### Diagnostic pathway (Figure 3)

518 TB suspects (31.1%) presented with known HIV status: 363 patients with a documented HIV positive test and 155 patients with a documented negative test of less than a year old. HIV testing was performed in 1124 TB suspects (98.0%) with unknown HIV status, in 6 patients a test was not asked and 17 patients refused to be tested. 79 (7.0%) of the TB suspects with unknown HIV status were HIV-positive. Sputum microscopy was performed in all but 34 TB suspects (98.0%). A chest X-ray was requested for 400 out of the 412 HIV-positive smear-negative TB suspects (97.1%). Samples for sputum culture were requested for 405 HIV-positive smear-negative TB suspects. 13 culture results were unavailable.

**Figure 3:**
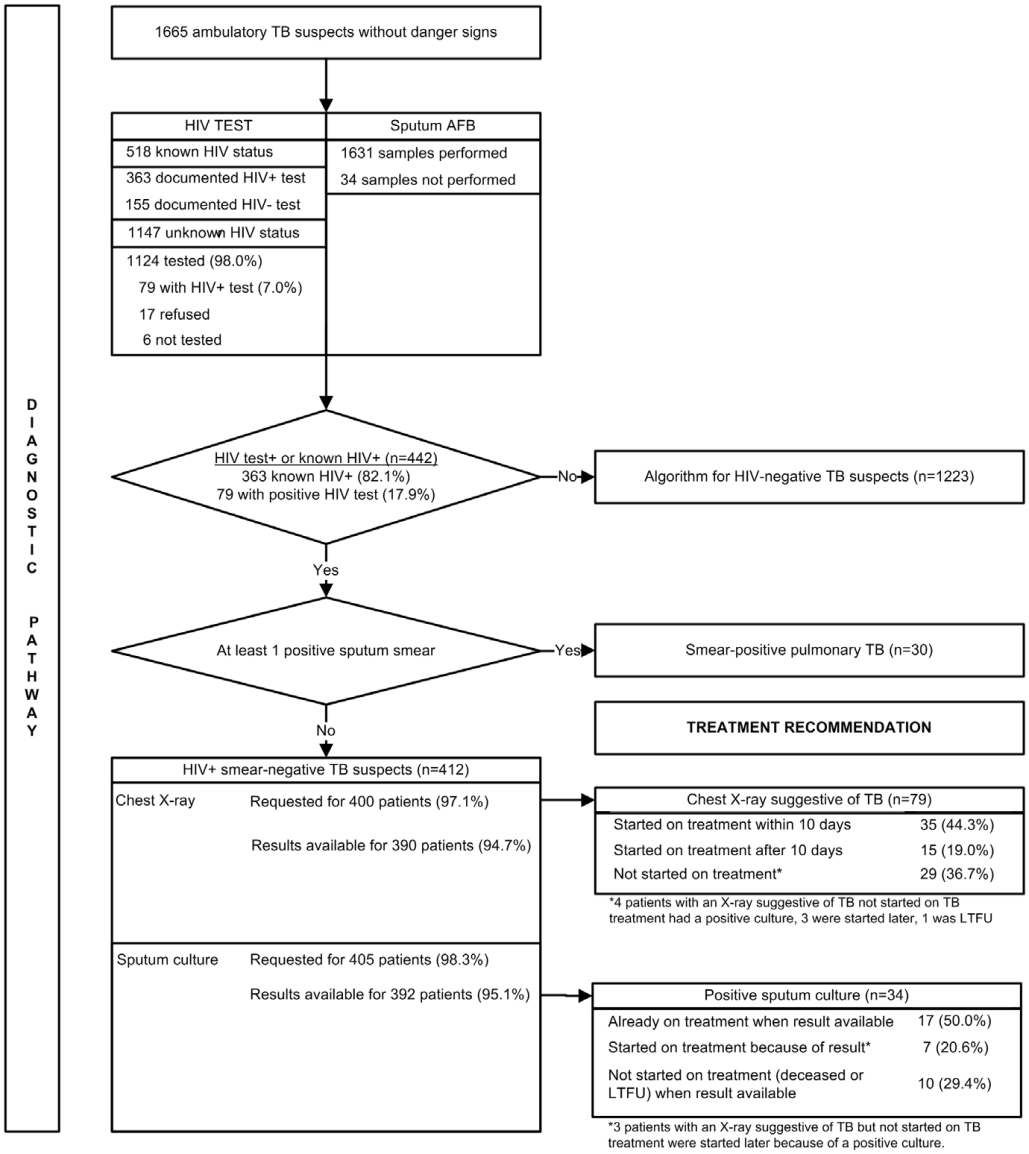
Operational performance of the WHO 2007 algorithm for the diagnosis of tuberculosis in ambulatory HIV-positive patients (diagnostic pathway and treatment recommendation).

#### Treatment recommendation (Figure 3)

The chest X-ray result was considered by the treating physician suggestive of TB in 79 smear-negative TB suspects (62 suspects with “cough > 2 weeks” and 17 with “no cough or cough <2 weeks”). Only 35 (44.3%) were started on TB treatment within 10 days, 15 (19.0%) were started on TB treatment after 10 days, and 29 (36.7%) were not started on TB treatment. The sputum culture was positive in 34 of the 392 smear-negative TB suspects for which results were available. 17 patients (50%) were already on treatment when the result came, 7 (20.6%) were started on treatment thanks to the culture result and 10 (29.4%) were not started on treatment despite the culture results, because the patients had died or were lost to follow-up by the time the result was available.

#### Incremental yield (Table 2)

Among the 105 patients who started TB treatment 30 were detected because of a positive smear, an additional 53 were treated because of a suggestive chest X-ray, 18 (17.1%) more because of clinical presentation or abdominal ultrasound suggestive of TB and only 4 (3.8%) because of positive culture. Among the 34 confirmed smear-negative TB patients, about forty percent (14/34) were diagnosed by chest X-ray, about 20% more by abdominal ultrasound or clinical suspicion and finally also forty percent by culture only (14/34).

**Table 2:** Incremental yield of different examinations in HIV-positive patients started on TB treatment and in patients with confirmed TB (reference standard: positive sputum culture).

	All patients who started TB treatment (n = 105)	Smear-negative TB suspects with confirmed TB diagnosis (n = 34)
**Positive smear**(at least 1 positive sputum smear)	30 (28.6%)	NA
**Chest X-ray suggestive of TB**	+53 (50.5%)*	+14 (41.2%)
**Clinical assessment suggestive of TB**(clinical suspicion or abdominal ultrasound)	+18 (17.1%)	+6 (17.6%)
**Positive culture**	+4 (3.8%)	+14 (41.2%)¶

*3 patients with an X-ray suggestive of TB but not started on TB treatment were started later because of a positive culture. 1 patient with an X-ray suggestive of TB but not started on TB treatment and a positive culture was LTFU.

¶ Of the 14 patients diagnosed by sputum culture only, only 4 were started on TB treatment.

The median time to detection among these 14 additional TB patients was 66.5 days (IQR: 56–70 days). As a result of the long detection time, only 4 of those 14 patients started TB treatment after a median time of 110 days (range: 73–147 days), 2 patients had died and 8 were LTFU.

### Diagnostic accuracy of the WHO 2007 algorithm for smear-negative TB

**Figure 4:**
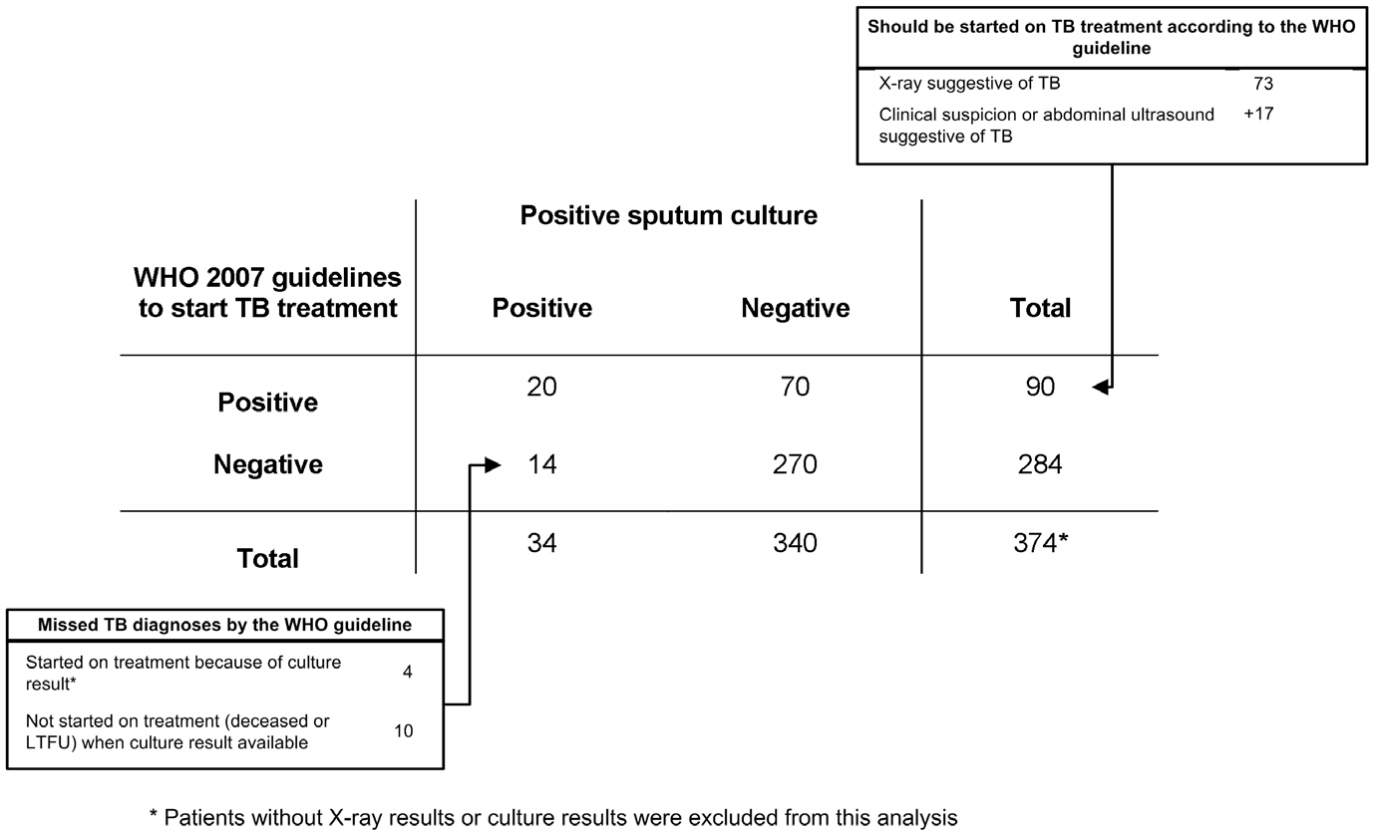
Diagnostic performance of the WHO guideline in the smear-negative cohort of TB suspects (reference: positive sputum culture).

Considering only the smear-negative cohort of TB suspects (n = 374), the diagnostic algorithm had a sensitivity of 58.8% (95%CI: 42.2%–73.6%) and a specificity of 79.4% (95%CI: 74.8%–82.4%) using culture positivity as reference standard. (**Table 3** **,** **Figure 4**) There was no statistically significant heterogeneity of the observed sensitivities and specificities of the diagnostic algorithm between the different sites.

If patients with an abnormal X-ray (irrespective whether suggestive of TB or otherwise) would have been started on TB treatment, the sensitivity would have increased to 64.7% (95%CI: 47.9%–78.5%) while the specificity would have decreased to 72.9% (95%CI: 68.0%–77.4) (data not shown).

**Table 3:** Diagnostic performance of the WHO 2007 algorithm in the smear-negative cohort of TB suspects, depending on inclusion criteria (reference: positive sputum culture).

	HIV+TB suspects* n (%)	Cough > 2 weeks n (%)	No cough or cough <2 weeks¶ n (%)
**Total, n (%)**	374 (100.0)	258 (69.0)	116 (31.0)
**Nr of confirmed TB cases (1 culture +)**	34	23	11
**Sensitivity, % (95% CI)**	58.8 (42.2–73.6)	73.9 (53.5–87.5)	27.3 (9.7–56.6)
**Specificity, % (95% CI)**	79.4 (74.8–82.4)	78.7(73.1–82.5)	81.0 (72.4–87.3)
**PPV, % (95% CI)**	22.2 (14.9–31.8)	25.4 (16.5–36.9)	13.0 (4.5–32.1)
**NPV, % (95% CI)**	95.1 (91.9–97.0)	96.9 (93.3–98.6)	91.4 (83.9–95.6)

CI: Confidence interval; PPV: Positive predictive value; NPV: Negative predictive value

*Patients without X-ray results or culture results were excluded from this analysis.

¶ Patients with no cough or cough <2 weeks had other inclusion criteria.

### Inter-observer agreement for chest X-rays

For the assessment of agreement between the readers, 349 chest X-ray reports were available. The inter-observer agreement was good for distinction between normal and abnormal X-rays (κ ranging from 0.62 to 0.72), but only fair for distinction between TB and non-TB (κ ranging from 0.28 to 0.42) (data not shown).

5 of the 79 chest X-rays suggestive of TB by the site physician were not available for interpretation by the 2 expert radiologists. Of the remaining 74 chest X-rays that were reported as suggestive of TB by the treating physician, only 48 (64.9%) and 42 (56.8%) were classified as suggestive of TB by the expert radiologists.

### TB categories and treatment outcomes

**Figure 5:**
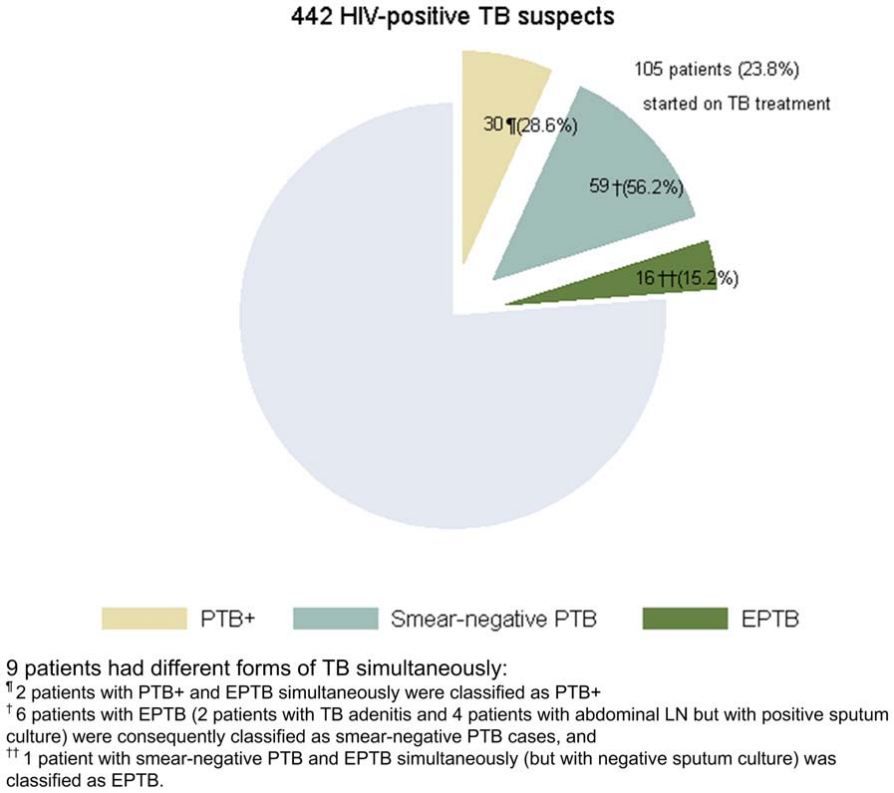
Distribution of TB categories among HIV-positive patients started on TB treatment.

TB treatment was started in 105 HIV-positive patients (**Figure 5**). Fifty-nine patients (56.2%) were categorized as smear-negative PTB, 16 (15.2%) as EPTB and 30 patients (28.6%) as smear-positive PTB. The patients with presumed EPTB were diagnosed by chest X-ray (miliary TB), abdominal ultrasound (enlarged abdominal lymph nodes) and by clinical examination (TB adenitis) resulting in short treatment delays.

The median time to TB treatment initiation was 5 days (IQR: 2–13 days), ranging from 2 days (IQR: 1–11.5 days) for EPTB, over 2.5 days (IQR: 1–4 days) for smear-positive PTB to 9 days (IQR: 3–17 days) for smear-negative PTB.

Seventy five patients (71.4%) completed treatment or were cured, 26 (24.8%) defaulted or were LTFU, 3 (2.8%) died, and 1 (1.0%) patient was categorized as treatment failure.

## Discussion

Our study evaluated the operational performance of the WHO 2007 guideline for the diagnosis of TB in patients considered as TB suspects in an HIV-prevalent setting. The WHO algorithm needed validation in low-resource settings that are meant to implement the guidelines. This brings about methodological difficulties as it is problematic to create a research environment in these circumstances allowing for a methodologically correct evaluation of diagnostic accuracy of the algorithm.

Our research indicates that the acceptance rate for HIV testing by TB suspects is high (98.0%) compared to other settings [24], and compliance with the diagnostic flowchart by health staff was acceptable. Although we did not compare the treatment delay with historical data from the same setting, we think the delay is reduced when comparing with data from literature on older algorithms. [25] Taking into account that HIV-positive smear-negative TB patients frequently do not receive a diagnosis for several months because of delays due to additional diagnostic testing or antibiotic treatment trials [26], the observed treatment delay of 5 days seems reasonable. The treatment delay would have been further reduced if compliance with the guideline in terms of faster treatment initiation were better. Only about half of patients with a smear-negative result who had a chest X-ray suggestive of TB were started on TB treatment without further delay. This contrasts with the readiness of the clinicians to start empirical treatment for presumed EPTB. Abdominal ultrasound identified 21% of the confirmed TB diagnosis in the setting where ultrasound was available, confirming the usefulness of abdominal ultrasound as a diagnostic test for TB in HIV-positive patients. [27]

Our research indicates that the sensitivity of the 2007 WHO guideline to diagnose smear-negative TB is acceptable: 58.8% (95%CI: 42.2%–73.6%) of patients with confirmed TB were identified by the diagnostic algorithm for smear-negative TB suspects. Although somehow disappointing, this represents a significant increase in early diagnosis compared to the previous guidelines and would lead to faster treatment in a significant proportion of patients if guidelines were implemented as recommended.

Sputum culture resulted in an additional yield of TB diagnoses but this was offset by the delay inherent to solid culture methods and the logistic constraints to trace patients post-hoc. About 70% of the TB patients identified additionally by culture alone had died or were LTFU when the results finally were available. Considering the poor prognosis of untreated smear-negative TB in HIV-positive patients we can assume that the LTFU patients have died, which would result in a ‘corrected’ mortality of about 10%. Liquid culture, as recommended by WHO [28] for high burden TB countries, would help to shorten the detection time and probably the mortality, but was not available in Cambodia at the time of the study. Regular transport of samples and results to and from the central laboratory are additional operational/logistical challenges for both methods.

We observed a good agreement for distinguishing normal from abnormal X-rays in all X-rays, but only a fair agreement for distinction between TB and non-TB. Chest X-rays are an important component of the algorithm, and adequate training should receive proper attention. But taking into account the difficulties in interpreting X-ray reading in HIV-positive patients [29], [30], the algorithm could be further simplified by commencing TB treatment in any HIV positive patient with an abnormal X-ray. This would increase the sensitivity of the algorithm in HIV-positive TB suspects to 64.7% (plus 5.9%) but at a cost of a decreased specificity of 72.9% (minus 6.5%).

The diagnostic means in this setting did not allow for a gold standard definition of EPTB, which is a weakness of the study. Moreover, our study is not assessing the sensitivity of the screening questions to define a TB suspect. As such, the diagnostic performance of the overall algorithm (both screening and diagnostic) was not assessed. Sputum smear microscopy identified a few smear-positive pulmonary tuberculosis cases among patients who did not present with cough > 2 weeks. If we would have used a more sensitive screening approach for PTB with a combination of symptoms as entry point for PTB (any cough, any fever or night sweats, or weight loss [31]–[34] without specifying the duration of cough as Cain et al. [35] suggest, these patients would likely have ended up in the PTB track. In our study the sensitivity of the guideline to diagnose TB in smear-negative TB suspects with other inclusion criteria than cough >2weeks is low: only 27.3% of patients with positive sputum culture were detected by X-ray, clinical suspicion or abdominal ultrasound. This further underscores the need to either revise the screening questions for PTB and include cough of any duration, or to do sputum cultures in al TB suspects, not only the ones coughing for more than 2 weeks.

Our study has some other limitations: we used solid culture as reference standard for detecting pulmonary and extrapulmonary TB. Culturing multiple specimens on liquid media would have detected more confirmed TB cases [36], [37], and could affect the sensitivity and specificity of the diagnostic algorithm among HIV-positive TB suspects.

The guidelines are meant for settings where the HIV prevalence exceeds 1% in the general population or 5% among the TB cases. In this study, 26.5% of the TB suspects were HIV-positive. However the HIV prevalence varied greatly across sites: from 2.7% at CENAT to 55.4% at SHCH. This highlights the need to identify within low HIV-prevalent countries the settings where the guideline should be implemented.

The heterogeneity among the sites might also have contributed to differences in delay of presentation of TB suspects at the study sites. Nevertheless we did not find a statistically significant difference in the diagnostic performance of the diagnostic algorithm between the study sites. However, due to the small number of confirmed TB cases (n = 34) the study lacks power to examine this interaction.

Our study provides evidence that the application of the WHO guideline, based on expert opinion, is feasible in the field. However, several barriers for better patient outcomes exist: solid culture needs to be replaced by liquid culture, sensitivity in smear-negative TB is still relatively low and more sensitive rapid diagnostic tests are needed such as the Xpert MTB/RIF assay. [38]

Additional qualitative research among health care workers could be useful to understand the hesitance to start patients on TB treatment. Endorsement of the results by WHO and national TB programs is important in order to remove perceived barriers to implementation by practitioners in the field. [39]

### Conclusions

Whilst awaiting easy point-of-care rapid diagnostic tests for TB disease, diagnostic algorithms have a role to play. The diagnostic accuracy of the 2007 WHO guideline to diagnose smear-negative TB is acceptable. HIV-testing in all TB suspects is acceptable to the patient and reduces delay in treatment if the recommendations are implemented. There is however reluctance to comply with the guideline in terms of immediate treatment initiation.

## References

[1] WHO (2009). Global Tuberculosis Control: a short update to the 2009 report.. http://www.who.int/tb/publications/global_report/2009/update/tbu_9.pdf.

[2] WHO (2009). Global Tuberculosis Control 2009.. http://www.who.int/tb/publications/global_report/2009/pdf/full_report.pdf.

[3] National AIDS Authority (2008). UNGASS Country Progress Report Cambodia.. http://data.unaids.org/pub/Report/2008/cambodia_2008_country_progress_report_en.pdf.

[4] Ministry of Health Cambodia (2008). Tuberculosis Report.

[5] Corbett EL, Watt CJ, Walker N, Maher D, Williams BG (2003). The growing burden of tuberculosis: global trends and interactions with the HIV epidemic.. Arch Intern Med.

[6] Colebunders R, Bastian I (2000). A review of the diagnosis and treatment of smear-negative pulmonary tuberculosis.. Int J Tuberc Lung Dis.

[7] Harries AD, Nyangulu DS, Kang'ombe C, Ndalama D, Glynn JR (1998). Treatment outcome of an unselected cohort of tuberculosis patients in relation to human immunodeficiency virus serostatus in Zomba Hospital, Malawi.. Trans R Soc Trop Med Hyg.

[8] Hargreaves NJ, Kadzakumanja O, Whitty CJ, Salaniponi FM, Harries AD (2001). ‘mear-negative’ pulmonary tuberculosis in a DOTS programme: poor outcomes in an area of high HIV seroprevalence.. Int J Tuberc Lung Dis.

[9] Mukadi YD, Maher D, Harries A (2001). Tuberculosis case fatality rates in high HIV prevalence populations in sub-Saharan Africa.. AIDS.

[10] Banda H, Kang'ombe C, Harries AD, Nyangulu DS, Whitty CJ (2000). Mortality rates and recurrent rates of tuberculosis in patients with smear-negative pulmonary tuberculosis and tuberculous pleural effusion who have completed treatment.. Int J Tuberc Lung Dis.

[11] Trinh TT, Shah NS, Mai HA, Do TN, Duong T (2007). HIV-associated TB in An Giang Province, Vietnam, 2001-2004: epidemiology and TB treatment outcomes.. PLoS ONE.

[12] Cain KP, Kanara N, Laserson KF, Vannarith C, Sameourn K (2007). The epidemiology of HIV-associated tuberculosis in rural Cambodia.. Int J Tuberc Lung Dis.

[13] Cain KP, Anekthananon T, Burapat C, Akksilp S, Mankhatitham W (2009). Causes of death in HIV-infected persons who have tuberculosis, Thailand.. Emerg Infect Dis.

[14] Getahun H, Harrington M, O'Brien R, Nunn P (2007). Diagnosis of smear-negative pulmonary tuberculosis in people with HIV infection or AIDS in resource-constrained settings: informing urgent policy changes.. Lancet.

[15] Lucas SB, De Cock KM, Hounnou A, Peacock C, Diomande M et al (1994). Contribution of tuberculosis to slim disease in Africa.. BMJ.

[16] Ansari NA, Kombe AH, Kenyon TA, Hone NM, Tappero JW (2002). Pathology and causes of death in a group of 128 predominantly HIV-positive patients in Botswana, 1997-1998.. Int J Tuberc Lung Dis.

[17] Greenberg AE, Lucas S, Tossou O, Coulibaly IM, Coulibaly D (1995). Autopsy-proven causes of death in HIV-infected patients treated for tuberculosis in Abidjan, Cote d'Ivoire.. AIDS.

[18] Cohen T, Murray M, Wallengren K, Alvarez GG, Samuel EY (2010). The prevalence and drug sensitivity of tuberculosis among patients dying in hospital in KwaZulu-Natal, South Africa: a postmortem study.. PLoS Med.

[19] Siddiqi K, Lambert ML, Walley J (2003). Clinical diagnosis of smear-negative pulmonary tuberculosis in low-income countries: the current evidence.. Lancet Infect Dis.

[20] WHO (2007). Improving the diagnosis and treatment of smear-negative pulmonary and extrapulmonary tuberculosis among adults and adolescents: Recommendations for HIV-prevalent and resource-constrained settings.. http://whqlibdoc.who.int/hq/2007/WHO_HTM_TB_2007.379_eng.pdf.

[21] WHO (2003). Treatment of tuberculosis: Guidelines for national programmes.. http://whqlibdoc.who.int/hq/2003/WHO_CDS_TB_2003.313_eng.pdf.

[22] Landis JR, Koch GG (1977). The measurement of observer agreement for categorical data.. Biometrics.

[23] WHO (2010). Treatment of tuberculosis: Guidelines for national programmes.. http://whqlibdoc.who.int/publications/2010/9789241547833_eng.pdf.

[24] Deribew A, Negussu N, Kassahun W, Apers L, Colebunders R (2010). Uptake of provider-initiated counselling and testing among tuberculosis suspects, Ethiopia.. Int J Tuberc Lung Dis.

[25] Sreeramareddy CT, Panduru KV, Menten J, Van den EJ (2009). Time delays in diagnosis of pulmonary tuberculosis: a systematic review of literature.. BMC Infect Dis.

[26] Perkins MD, Cunningham J (2007). Facing the crisis: improving the diagnosis of tuberculosis in the HIV era.. J Infect Dis.

[27] Sculier D, Vannarith C, Pe R, Thai S, Kanara N (2010). Performance of abdominal ultrasound for diagnosis of tuberculosis in HIV-infected persons living in Cambodia.. J Acquir Immune Defic Syndr.

[28] WHO (2009). Strategic and Technical Advisory group for Tuberculosis.. http://www.who.int/tb/events/stag_report_2007.pdf.

[29] Johnson JL, Vjecha MJ, Okwera A, Hatanga E, Byekwaso F (1998). Impact of human immunodeficiency virus type-1 infection on the initial bacteriologic and radiographic manifestations of pulmonary tuberculosis in Uganda. Makerere University-Case Western Reserve University Research Collaboration.. Int J Tuberc Lung Dis.

[30] Post FA, Wood R, Pillay GP (1995). Pulmonary tuberculosis in HIV infection: radiographic appearance is related to CD4+ T-lymphocyte count.. Tuber Lung Dis.

[31] Reid MJ, Shah NS (2009). Approaches to tuberculosis screening and diagnosis in people with HIV in resource-limited settings.. Lancet Infect Dis.

[32] Bassett IV, Wang B, Chetty S, Giddy J, Losina E (2010). Intensive tuberculosis screening for HIV-infected patients starting antiretroviral therapy in Durban, South Africa.. Clin Infect Dis.

[33] Kranzer K, Houben RM, Glynn JR, Bekker LG, Wood R (2010). Yield of HIV-associated tuberculosis during intensified case finding in resource-limited settings: a systematic review and meta-analysis.. Lancet Infect Dis.

[34] Getahun H, Kittikraisak W, Heilig CM, Corbett EL, Ayles H (2011). Development of a Standardized Screening Rule for Tuberculosis in People Living with HIV in Resource-Constrained Settings: Individual Participant Data Meta-analysis of Observational Studies.. PLoS Med.

[35] Cain KP, McCarthy KD, Heilig CM, Monkongdee P, Tasaneeyapan T (2010). An algorithm for tuberculosis screening and diagnosis in people with HIV.. N Engl J Med.

[36] Wilson D, Nachega J, Morroni C, Chaisson R, Maartens G (2006). Diagnosing smear-negative tuberculosis using case definitions and treatment response in HIV-infected adults.. Int J Tuberc Lung Dis.

[37] Monkongdee P, McCarthy KD, Cain KP, Tasaneeyapan T, Dung NH (2009). Yield of Acid-fast Smear and Mycobacterial Culture for Tuberculosis Diagnosis in People with HIV.. Am J Respir Crit Care Med.

[38] Boehme CC, Nabeta P, Hillemann D, Nicol MP, Shenai S (2010). Rapid molecular detection of tuberculosis and rifampin resistance.. N Engl J Med.

[39] Ralph AP, Anstey NM, Kelly PM (2009). Tuberculosis into the 2010s: is the glass half full?. Clin Infect Dis.

